# Two pore domain potassium channels in cerebral ischemia: a focus on K_2P_9.1 (TASK3, KCNK9)

**DOI:** 10.1186/2040-7378-2-14

**Published:** 2010-07-20

**Authors:** Petra Ehling, Stefan Bittner, Nicole Bobak, Tobias Schwarz, Heinz Wiendl, Thomas Budde, Christoph Kleinschnitz, Sven G Meuth

**Affiliations:** 1 Westfaelische Wilhelms-University Muenster, Institute of Physiology I, Robert-Koch Str. 27a, 48149 Muenster, Germany; 2 University of Wuerzburg, Department of Neurology, Josef-Schneider Str. 11, 97080 Wuerzburg, Germany; 3 University of Muenster, Neurological Clinic - Inflammatory Disorders of the Central Nerveous System and Neurooncology, Mendelstr. 7, 48149 Muenster, Germany

## Abstract

**Background:**

Recently, members of the two-pore domain potassium channel family (K_2P _channels) could be shown to be involved in mechanisms contributing to neuronal damage after cerebral ischemia. K_2P_3.1^-/- ^animals showed larger infarct volumes and a worse functional outcome following experimentally induced ischemic stroke. Here, we question the role of the closely related K_2P _channel K_2P_9.1.

**Methods:**

We combine electrophysiological recordings in brain-slice preparations of wildtype and K_2P_9.1^-/- ^mice with an in vivo model of cerebral ischemia (transient middle cerebral artery occlusion (tMCAO)) to depict a functional impact of K_2P_9.1 in stroke formation.

**Results:**

Patch-clamp recordings reveal that currents mediated through K_2P_9.1 can be obtained in slice preparations of the dorsal lateral geniculate nucleus (dLGN) as a model of central nervous relay neurons. Current characteristics are indicative of K_2P_9.1 as they display an increase upon removal of extracellular divalent cations, an outward rectification and a reversal potential close to the potassium equilibrium potential. Lowering extracellular pH values from 7.35 to 6.0 showed comparable current reductions in neurons from wildtype and K_2P_9.1^-/- ^mice (68.31 ± 9.80% and 69.92 ± 11.65%, respectively). These results could be translated in an in vivo model of cerebral ischemia where infarct volumes and functional outcomes showed a none significant tendency towards smaller infarct volumes in K_2P_9.1^-/- ^animals compared to wildtype mice 24 hours after 60 min of tMCAO induction (60.50 ± 17.31 mm^3 ^and 47.10 ± 19.26 mm^3^, respectively).

**Conclusions:**

Together with findings from earlier studies on K_2P_2.1^-/- ^and K_2P_3.1^-/- ^mice, the results of the present study on K_2P_9.1^-/- ^mice indicate a differential contribution of K_2P _channel subtypes to the diverse and complex in vivo effects in rodent models of cerebral ischemia.

## Background

Although ischemic stroke represents a major health care problem with a high rate of permanent disability or even death, the underlying molecular mechanisms leading to neuronal death are still poorly understood [1]. However, ion channels which can influence basal cellular parameters are thought to play a major role within this context. Activation of potassium channels results in membrane hyperpolarization thereby decreasing neuronal activity and cell death under pathophysiological conditions. Additionally, K^+ ^channels (e.g. large conductance Ca^2+^-activated K^+ ^channels and ATP-sensitive K^+ ^channels [2,3]) might be neuroprotective as they counterbalance a prolonged harmful influx of Ca^2+ ^ions via different pathways including a reversal of the Na^+^/Ca^2+ ^antiporter and voltage-dependent Ca^2+ ^channels. Furthermore, an enhancement of the Mg^2+ ^block of NMDA receptors (N-methyl D-aspartate) in postsynaptic neurons [4] is thought to protect against glutamate excitotoxicity [5,6].

Concerning the recently identified family of two-pore domain potassium channels (K_2P _channels), several members have been shown to play a major role in critical conditions leading to cerebral ischemia. K_2P_2.1^-/- ^mice displayed significantly less neuronal survival rates in a model of cerebral ischemia [7]. These data were confirmed by the neuroprotective effect of several K_2P_2.1 channel activators (e.g. alpha linelonic acid or riluzole [8-10]). On the other hand, genetic depletion of another family member, namely K_2P_3.1, resulted in increased infarct volumes following transient or permanent middle cerebral artery occlusion (MCAO) [11,12]. Based on sequence homologies and similar biophysical properties, it was suggested that related channel family members might also be of importance under these circumstances. We challenged the role of K_2P_9.1 (TASK3; KCNK9) in a tMCAO model using previously described K_2P_9.1^-/- ^mice [13].

## Methods

### Slice preparation

Thalamic tissue slices including the dorsal lateral geniculate nucleus (dLGN) were prepared from 14 - 22 days old male C57BL/6 or K_2P_9.1^-/- ^mice [13] as described earlier [14]. Coronal sections were cut on a vibratome (Vibratome^®^, Series 1000 Classic, St. Louis, USA) in an ice-chilled solution containing (mM): Sucrose, 200; PIPES, 20; KCl, 2.5; NaH_2_PO_4_, 1.25; MgSO_4_, 10; CaCl_2_, 0.5; dextrose, 10; pH 7.35 adjusted with NaOH. Prior to the recording procedure, slices were kept submerged in artificial cerebrospinal fluid (ACSF, mM): NaCl, 125; KCl, 2.5; NaH_2_PO_4_, 1.25; NaHCO_3_, 24; MgSO_4_, 2; CaCl_2_, 2; dextrose, 10; pH adjusted to 7.35 by bubbling with a mixture of 95% O_2 _and 5% CO_2_.

### Electrophysiology

Slices were transferred in a recording chamber and thalamic neurons of the dLGN were visualized with a microscope equipped with infrared-differential interference contrast optics [15]. Whole-cell recording pipettes were fabricated from borosilicate glass (GT150T-10, Clark Electromedical Instruments, Pangbourne, UK; typical resistance 2-3 MΩ) and filled with an intracellular solution containing (in mM): K-gluconate, 88; K_3_-citrate, 20; NaCl, 10; HEPES, 10; MgCl_2_, 1; CaCl_2_, 0.5; BAPTA, 3; phosphocreatin, 15; Mg-ATP, 3; Na-GTP, 0.5. The internal solution was set to a pH value of 7.25 using KOH and an osmolarity of 295 mOsm/kg. Slices were continuously superfused with a solution containing NaCl 125 mM, KCl 2.5 mM, NaH_2_PO_4 _1.25 mM, HEPES 30 mM, MgSO_4 _2 mM, CaCl_2 _2 mM and dextrose 10 mM. Whole-cell patch-clamp recordings were measured from relay neurons of the dLGN with an EPC-10 amplifier (HEKA Elektronik, Lamprecht, Germany) and digitally analyzed using Pulse software (HEKA Elektronik; [16]). pH was adjusted to 7.35 or 6.0 with HCl. For divalent-cation-free conditions we switched from control solution to a solution containing 0 mM Mg^2+ ^and 0 mM Ca^2+^; the osmolality was kept constant at 305 mosmol kg^-1 ^by adding 4 mM NaCl [17]. All cells had a resting membrane potential negative to -65 mV, the access resistance was in the range of 5-15 MΩ and series resistance compensation of more than 40% was routinely used.

### Induction of cerebral ischemia

Animal experiments were approved by governmental agencies for animal research and conducted according to the recommendations for research in mechanism-driven basic stroke studies [18]. Focal cerebral ischemia was induced in 6-8 weeks old male C57BL/6 and K_2P_9.1^-/- ^mice [13] weighing 20-25 g by transient middle cerebral artery occlusion (tMCAO) as described previously [19,20]. Briefly, mice were anesthetized with 2.5% isoflurane (Abbott, Wiesbaden, Germany) in a 70% N_2_O/30% O_2 _mixture. Core body temperature was maintained at 37°C throughout surgery using a feedback-controlled heating device. Following a midline skin incision in the neck, the proximal common carotid artery and the external carotid artery were ligated and a standardized silicon rubber-coated 6.0 nylon monofilament (6021; Doccol Corp., CA, USA) was inserted and advanced via the right internal carotid artery to occlude the origin of the right MCA. The intraluminal suture was left *in situ *for 1 hour, respectively. Then animals were re-anesthetized and the occluding monofilament was withdrawn to allow reperfusion. After 24 hours neurological deficits were scored by two blinded investigators and quantified according to Bederson [21]: 0, no deficit; 1, forelimb flexion; 2, as for 1, plus decreased resistance to lateral push; 3, unidirectional circling; 4, longitudinal spinning; 5, no movement. For the gript test, the mouse was placed midway on a string between two supports and rated as follows: 0, falls off; 1, hangs onto string by one or both forepaws; 2, as for 1, and attempts to climb onto string; 3, hangs onto string by one or both forepaws plus one or both hindpaws; 4, hangs onto string by fore- and hindpaws plus tail wrapped around string; 5, escape (to the supports).

Laser doppler flowmetry (Moor Instruments, Axminster, United Kingdom) was used to monitor cerebral blood flow [22] in wildtype, K_2P_9.1^-/- ^and sham-treated animals (n = 4/group) before surgery (baseline), immediately after MCA occlusion, and 5 minutes after removal of the occluding monofilament (reperfusion). Cerebral perfusion did not differ between the groups at any time point (Additional File 1, Figure S1).

### Determination of infarct size

Mice were sacrificed 24 hours after tMCAO, respectively. Brains were quickly removed and cut in 2 mm thick coronal sections using a mouse brain slice matrix. The slices were stained with 2% 2,3,5-triphenyltetrazolium chloride (TTC; Sigma-Aldrich, St. Louis, MO) in PBS to visualize the infarctions. Planimetric measurements (ImageJ software, National Institutes of Health, Bethesda, MD) blinded to the treatment groups were used to calculate lesion volumes, which were corrected for brain edema as described [23,24].

### Statistical analysis

Electrophysiological data and results from the animal experiments were analyzed by a modified student's t test for small samples [25] or by a Bonferroni-corrected One-way ANOVA in case of multiple comparisons using PrismGraph 4.0 software (GraphPad Software, San Diego, CA) or Origin^® ^(Microcal). P-values < 0.05 were considered statistically significant.

## Results

### **Thalamic relay neurons as a model system of central nervous system neurons display electrophysiological properties indicative of currents through K_2P_9.1 channels**

**Figure 1:**
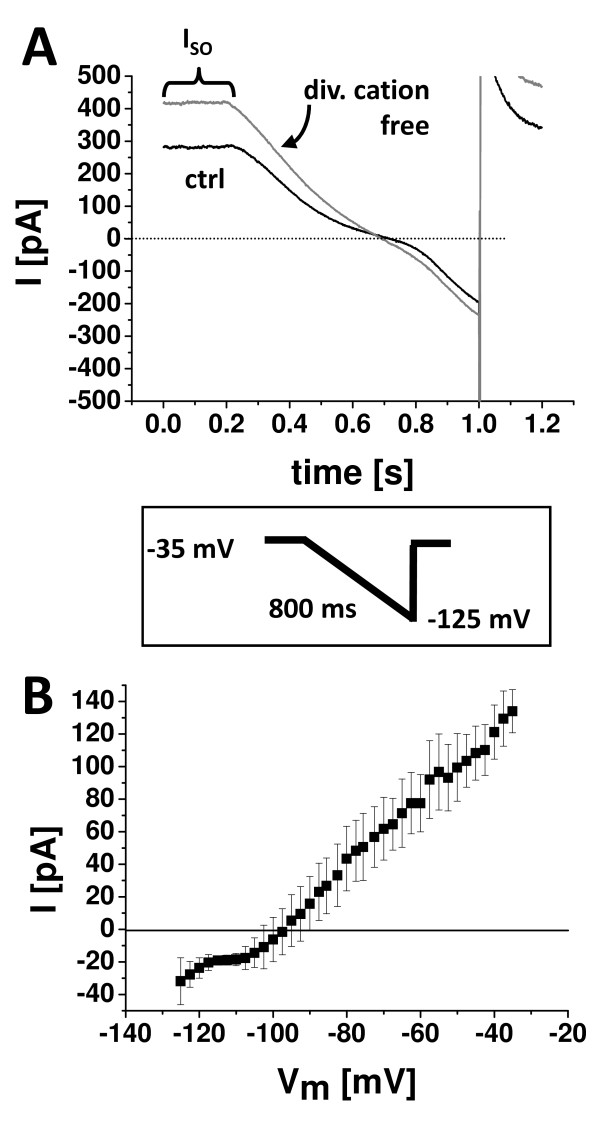
**Whole cell currents recorded from relay neurons in thalamic slice preparations show characteristics indicative for K_2P_9.1 channels**. (**A**) Mean ramp currents under control recording conditions (ctrl) and after removal of extracellular divalent cations (div. cation free). Inset: Currents were evoked by ramping the membrane potential from -35 mV to -125 mV over 800 ms. (**B**) *I-V *relationship of the divalent cation-sensitive current component shows characteristics indicative of TASK channels. I = current; I_SO _= standing outward current; V_M _= membrane potential.

K_2P_9.1-like currents have been demonstrated in a number of different central nervous system neurons [14,26-28]. As highly specific inhibitors for K_2P _channel subtypes are not available, different semi-selective blockers and experimental strategies to distinguish between these channels were established. Among them, extracellular reduction of divalent cations was introduced to increase potassium outward currents through K_2P_9.1 channels [17]. Current-voltage relationships (I/V) of the standing outward current of wildtype and K_2P_9.1^-/- ^mice were investigated by ramping the membrane potential from -35 mV to -125 mV over 800 ms (Fig. 1A, inset; [29,30]). Under control conditions a standing outward current (I_SO_) of 322.33 ± 30.20 pA was measured at -35 mV (Fig. 1A). Application of hyperpolarizing voltage ramps induced a complex current response. The wave form of this response was indicative for the contribution of current through outwardly rectifying TASK channels as well as inwardly rectifying K^+ ^channels (Fig. 1A, black trace). Removal of extracellular divalent cations resulted in a significant increase of I_SO _by 35.47 ± 9.59% compared to control conditions (n = 6, p = 0.007; Fig. 1A). Ramp responses revealed a clear increase in the outwardly rectifying component (Fig. 1A, gray trace). The current sensitive to administering cation-free conditions was calculated by numerical subtraction of control currents from currents recorded under cation-free conditions [14]. The I/V relationship of the cation-sensitive current was typical of TASK channels with a strong outward rectification and a reversal potential close to the expected potassium equilibrium potential (Fig. 1B; E_K _= -104 mV). These findings indicate a strong contribution of K_2P_9.1 channels to the I_SO _of thalamocortical (TC) neurons in wildtype mice.

### **Neurons from K_2P_9.1^-/- ^and wildtype animals show no significant differences upon extracellular acidification**

**Figure 2:**
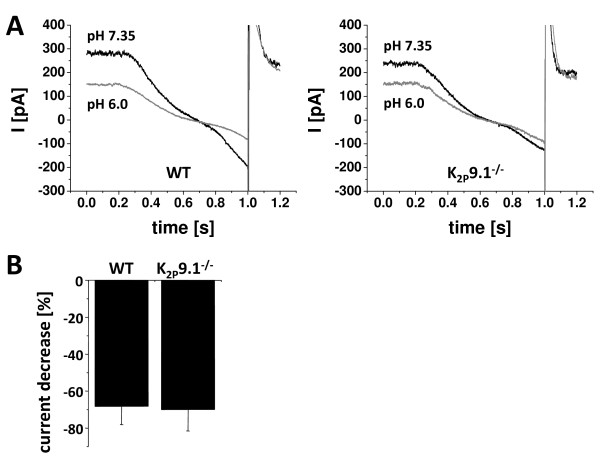
**pH dependence of the standing outward currents in K_2P_9.1^-/- ^neurons shows no difference compared to wildtype mice (pH 7.35 → pH 6.0)**. (**A**) Ramp currents under control conditions (pH 7.35) and after extracellular acidification (pH 6.0) in wildtype (WT; left panel) and K_2P_9.1^-/- ^mice (right panel). (**B**) Bar graph representation of current reduction after extracellular pH lowering in wildtype (WT) and K_2P_9.1^-/- ^mice.

Sensitivity to extracellular acidification is a hallmark of TASK channels and a reduction of the extracellular pH value can be typically observed under ischemic conditions. In a next experimental step we therefore mimicked cerebral ischemia by lowering the extracellular pH from control conditions (7.35) to 6.0. This maneuver resulted in a significant (p < 0.05) reduction of I_SO _amplitudes in both genotypes (Fig. 2A). The degree of I_SO _reduction was not different in wildtype (68.31 ± 9.80%) and K_2P_9.1^-/- ^neurons (69.92 ± 11.65%; n = 5; p = 0.91; Fig. 2B).

### **Genetic ablation of K_2P_9.1 channels trends to result in a not significant reduction of stroke development after tMCAO**

**Figure 3:**
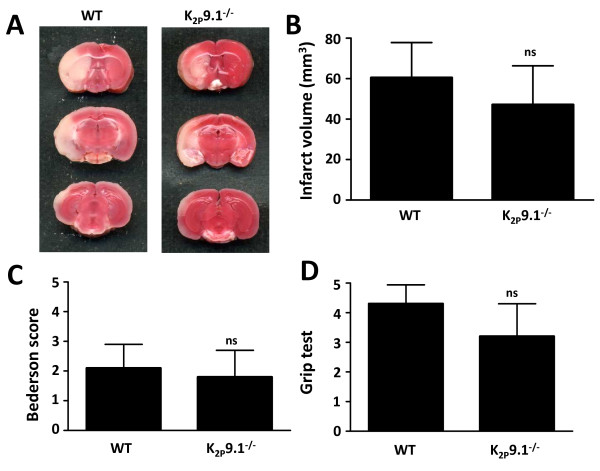
**Infarct volumes 24 h after 60 min MCA occlusion in wildtype and K_2P_9.1^-/- ^mice**. (**A**) Representative TTC-stained images of three corresponding coronal sections of control animals (WT) and K_2P_9.1^-/- ^mice. (**B**) Mean brain infarct volumes calculated from (A) (control group: n = 10; K_2P_9.1^-/- ^mice: n = 8). (**C**) Mean Bederson score and (**D**) grip test from the animals shown in (B). ns = not significant.

Stroke volumes of wildtype and K_2P_9.1^-/- ^mice were determined 24 hours after animals subjected to 60 min of tMCAO. Wildtype animals showed stroke volumes of 60.50 ± 17.31 mm^3 ^while K_2P_9.1^-/- ^mice displayed infarct areas of 47.10 ± 19.26 mm^3 ^(n = 10 and 8; p = 0.23; Fig. 3A and 3B). In accordance with this tendency towards none significantly smaller infarct sizes in K_2P_9.1^-/-^, no functionally relevant differences could be found for the Bederson score (WT: 1.83 ± 0.98; K_2P_9.1^-/-^: 2.14 ± 0.80; n = 6; p = 0.55; Fig. 3C) and the grip test (WT: 3.17 ± 1.13; K_2P_9.1^-/-^: 4.29 ± 0.64; n = 6; p = 0.09; Fig. 3D).

## Discussion

The results of the present study can be summarized as follows: (1) A pH- and divalent cation-sensitive I_SO _is present in TC neurons of the dLGN. (2) The divalent cation-sensitive component is characterized by outward rectification and a reversal potential close to the potassium equilibrium potential. (3) The I_SO _of neurons recorded from brain slices of K_2P_9.1^-/- ^mice and wildtype mice showed comparable pH-sensitivity during extracellular pH changes from 7.35 to 6.0. (4) In a model of cerebral ischemia, K_2P_9.1^-/- ^animals showed a tendency to reduced infarct volumes 24 hours after undergoing 60 min of tMCAO compared to wildtype controls although these results were not statistically significant. (5) It is concluded that K_2P_9.1-containing homodimeric and heterodimeric channels significantly contribute to I_SO _in TC neurons from wildtype mice and that K_2P_9.1 channels have only a minor impact on infarct volume and motor function following tMCAO compared to other members of the K_2P _channel family.

### **Contribution of TASK channel subtypes to I_SO _in TC neurons**

During development, the mouse thalamus is characterized by high K_2P_3.1 gene expression at P0 and displays moderate expression levels throughout postnatal stages [31]. K_2P_9.1 expression in many thalamic nuclei is rather moderate for all developmental stages but is strong in dLGN from P14 to adult stages. Functional TASK channels can be K_2P_3.1 homodimers, K_2P_9.1 homodimers, and K_2P_3.1/K_2P_9.1 heterodimers [32-35]. Although K_2P_3.1 and K_2P_9.1 show high sequence homology, they differ in their sensitivity to extracellular divalent cations (Mg^2+^, Ca^2+^) based on the presence of a glutamate residue at position 70 in K_2P_9.1 channels [17]. While the conductance of K_2P_3.1 homodimeric channels is unaffected, the conductance of K_2P_9.1 homodimeric and K_2P_3.1/K_2P_9.1 heterodimeric channels is strongly reduced in the presence of divalent cations [17,33]. Therefore the increase in I_SO _following removal of extracellular divalent cations which was found in cells from different rodent strain (Long Evans rats, wildtype mice, K_2P_3.1^-/- ^mice) point to the functional expression of K_2P_9.1 homodimeric and K_2P_3.1/K_2P_9.1 heterodimeric channels in TC neurons.

Homodimeric and heterodimeric TASK channels also differ in their pH-sensitivity. While K_2P_3.1/K_2P_9.1 channel constructs have a pH-sensitivity (pK approximately 7.3) in the physiological range which is closer to that of K_2P_3.1 channels (pK approximately 7.5) than K_2P_9.1 channels (pK approximately 6.8) [34]. In the present study no significant difference was found for the decrease in I_SO _amplitude when the pH was shifted to a value of 6.0. Therefore the pH- and divalent cation- sensitivities of native TASK-like currents in TC neurons is best represented by K_2P_3.1/K_2P_9.1 heterodimeric channels. However, additional modulators (isoflurane, Zn^2+^, ruthenium red) have to be tested to get more indications for the ratio of homodimeric to heterodimeric TASK channels in these neurons.

### **The role of TASK channel subtypes in ischemic insults**

It has been shown before that K_2P_3.1^-/- ^mice reveal larger tMCAO lesions in comparison to wildtype mice probably through a combination of direct neuronal effects and due to blood pressure/aldosterone effects [11,12]. Based on its physiogical properties and expression pattern, it seemed reasonable to expect an at least similar phenotype of K_2P_9.1^-/- ^mice compared to K_2P_3.1^-/- ^mice. The reason for the unexpected results presented here remains unclear but may involve one or more of the following considerations: (1) The cell type-specific expression and the exact conditions of the cellular environment of TASK channels have to be taken into account [36]. (2) Compensatory mechanisms, e.g. upregulation of other K_2P _channel family members, differences in oxygen sensitivity or yet unknown K_2P _channel properties may play a role. (2) In GABAergic interneurons of the entorhinal cortex membrane depolarization mediated by inhibition of K_2P_9.1 channels induce an increase in action potential firing [37]. In consequence, an increase in the release of GABA by interneurons results in a decrease in pyramidal cell activity thereby limiting the injurious effects of ischemia. Assuming that this type of network interaction is found in brain regions affected by tMCAO, the neuroprotection by K_2P_9.1^-/- ^channels is missing in knock out mice. (4) Gender differences should be taken into account [12]. (5) It should also be kept in mind that ischemic conditions may also affect a variety of other target structures including several ion channel, e.g. TRPV1 or ASICs as well as NMDA receptors [36].

To unravel the complex scenario of cerebral ischemia and to define the exact functional contribution of a particular K_2P _channel family member, further pharmacological and genetic tools are warranted, e.g. cell-type specific or conditional K_2P_3.1^-/-^, K_2P_9.1^-/- ^or K_2P_10.1^-/- ^mice. Especially the development of highly specific channel inhibitors or activators might open up the opportunity to procede these research efforts.

Taken together, results from K_2P_2.1^-/- ^(enhancement of ischemic damage [7]), K_2P_3.1^-/- ^(increase in infarct volumes [11,12]) and K_2P_9.1^-/- ^(no significant change ([12] or a tendency towards none significant reduced infarkt volumes: this work)) mice underline the fact that there are differential effects of different K_2P _channel subtypes on cerebral ischemia, not allowing to reason a uniform influence of this intriguing channel family on stroke formation.

## Competing interests

The authors declare that they have no competing interests.

## Authors' contributions

All authors have read and approved the final manuscript. PE, SB, NB and TB performed and analyzed the electrophysiological recordings. CK and TS operated the animals, assessed the functional scores and interpreted the data. HW, CK, TB, SB and SGM conceived the experiments, analyzed data, funded the project and wrote the manuscript.

## Supplementary Material

Additional file 1 **Figure S1 - rCBF does not differ between wildtype mice and K_2P_9.1^-/- ^mice**. Determination of regional cerebral blood flow (rCBF) using Laser Doppler flowmetry before the occlusion of the middle cerebral artery (baseline), 10 min after the occlusion (ischemia) and again 10 min after the removal of the filament (reperfusion) in wildtype mice and K_2P_9.1^-/- ^mice (n = 3/group). No significant differences in rCBF were observed between the two groups. One-way ANOVA, Bonferroni post hoc test. Click here for file
