# Correction: Two pore domain potassium channels in cerebral ischemia: a focus on K2P9.1 (TASK3, KCNK9)

**DOI:** 10.1186/2040-7378-5-3

**Published:** 2013-02-04

**Authors:** Petra Ehling, Stefan Bittner, Nicole Bobak, Tobias Schwarz, Heinz Wiendl, Thomas Budde, Christoph Kleinschnitz, Sven G Meuth

**Affiliations:** 1

## 

It has come to our attention that there is an error in the Acknowledgements section of our article [1]. The Acknowledgements section should read:

We are grateful to Ms. Melanie Glaser for excellent technical assistance. We are grateful to Douglas A. Bayliss for providing K_2P_9.1^-/-^ animals. This work was supported by the Interdisziplinäres Zentrum für klinische Forschung (IZKF A-54-1) to SGM and HW, the DFG (SFB 581, TP: A10) to SGM and DFG K2P-Forschergruppe 1086 (BU1019/9-1 to TB and ME3283/1-1 to SGM) and the Else-Kröner-Fresenius Foundation (2010_A95 to CK and SGM). PE was a member of the Otto-Creutzfeldt-Center for Cognitive and Behavioral Neuroscience Muenster (OCC).
