# Supplementary figures and images for: Two pore domain potassium channels in cerebral ischemia: a focus on K2P9.1 (TASK3, KCNK9)
Source: Exp Transl Stroke Med. 2010 Jul 20;2:14. doi: 10.1186/2040-7378-2-14 (PMC2912796; doi:10.1186/2040-7378-2-14)

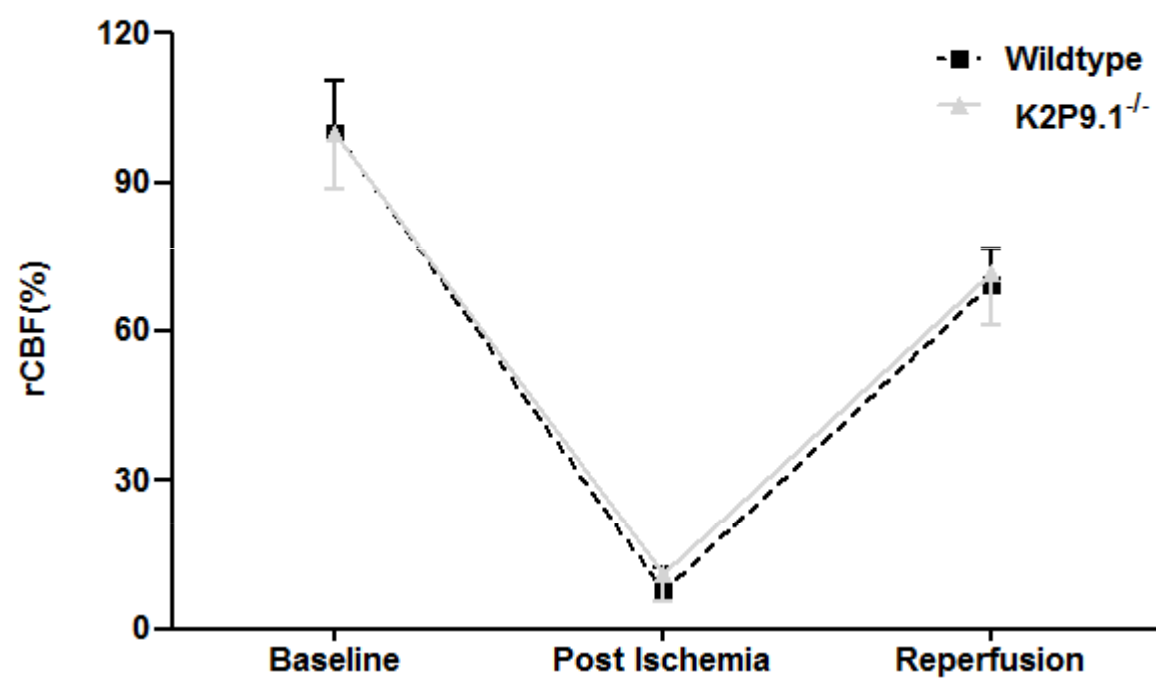

Supplement: Additional file 1 — Figure S1 - rCBF does not differ between wildtype mice and K2P9.1-/- mice. Determination of regional cerebral blood flow (rCBF) using Laser Doppler flowmetry before the occlusion of the middle cerebral artery (baseline), 10 min after the occlusion (ischemia) and again 10 min after the removal of the filament (reperfusion) in wildtype mice and K2P9.1-/- mice (n = 3/group). No significant differences in rCBF were observed between the two groups. One-way ANOVA, Bonferroni post hoc test. [file 2040-7378-2-14-S1.PDF]
